# Supplementary material for: Pet dogs’ Behavioural Reaction to Their Caregiver’s Interactions with a Third Party: Join in or Interrupt?
Source: Animals (Basel). 2022 Jun 18;12(12):1574. doi: 10.3390/ani12121574 (PMC9219478; doi:10.3390/ani12121574)
Supplement: Supplementary file 1 [file animals-12-01574-s001.zip › 220606_Karl et al_Pet dogsí» behavioural reaction to their caregiverí»s interactions with a third party_join in or interrupt_Supplem Material.pdf]

Pet dogs' behavioural reaction to their caregiver's interactions with a third party: join in or interrupt?

Sabrina Karl[1], Kristina Anderle[1], Christoph J. Völter[1] & Zsófia Virányi[1]

[1]Comparative Cognition, Messerli Research Institute, University of Veterinary Medicine Vienna, Medical University of Vienna, University of Vienna, 1210 Vienna, Austria

Supplementary Material

Table S1. Questions of jealousy caregiver questionnaire and summary of descriptive caregivers' reports (N=102).

Question	Caregiver report	Percentage
1) Does your dog react jealous/ jealousy-like towards other dogs?	Yes.	69.61 %
	No.	30.39% (including 8% stated some jealousy-like behaviours)
2) If your dog reacts jealous/ jealousy-like, which behaviours are shown?	Dog squeezing in-between the human-dog interaction, trying to separate the caregiver from the other dog, reacting aggressive by growling, snarling, barking, launching and snapping at the other dog, trying to regain the caregiver's attention and affection, e.g., by jumping at or licking him/ her, showing	69.61 %

	displacement behaviours like play bowing and sniffing, stress induced signals such as lip licking, whining, yawning, screaming or displaying piloerection and tail-wagging.	
3) When does your dog display jealous/ jealousy-like behaviours?	When the caregiver petted, praised, trained, played with or talked to another dog or when the other dog approached or was too close to the caregiver.	69.61 %
4) Where or in which situations does your dog display jealous/ jealousy-like behaviours?	At home.	36.27 %
	In dog zones.	15.69 %
	During dog walk.	15.69 %
	Location-independent.	6.86 %
	Differed a lot, also dog- and human-dependent.	6.86 %
	The other dog's home.	1.96 %
	In dog school.	0.98 %

Table S2. Results of mixed ordinal regression of behavioural ratings in the Introduction and Interaction phase.

	Estimate	SE	χ^2	df	P
Phase	-1.515	0.321	26.951	1	<0.001
Treatment[1]	-1.142	0.523			
Human[2]	-0.037	0.513			
Sex[3]	-0.236	0.365	0.417	1	0.519
Age[4]	-0.559	0.202	8.534	1	0.003
Treatment[1]:Human[2]	1.558	0.742	4.505	1	0.034

Notes: reference categories: [1]vet check; [2]caregiver; [3]female; [4]centered and standardized to a mean of zero and a standard deviation of 1.

Table S3. Results of binomial GLM of dogs' first approach of fake dog response in the Reaction phase.

	Estimate	SE	95% CI		χ^2	df	P
(Intercept)	-0.176	0.462	-1.103	0.730			
Human[1]	1.198	0.528	0.199	2.293	5.580	1	0.018
Treatment[2]	0.834	0.508	-0.147	1.860	2.771	1	0.096
Age[3]	-0.034	0.264	-0.540	0.473	0.016	1	0.898
Sex[4]	0.477	0.509	-0.511	1.502	0.889	1	0.346

Notes: reference categories: [1]caregiver; [2]vet check; [4]female; [3]centered and standardized to a mean of zero and a standard deviation of 1.

Table S4. Results of binomial GLM of dogs' friendly interaction with fake dog response in the Reaction phase.

	Estimate	SE	95% CI		χ^2	df	P
(Intercept)	-1.581	0.531	-2.714	-0.605			
Human[1]	-1.076	0.536	-2.185	-0.058	4.301	1	0.038
Treatment[2]	1.459	0.564	0.404	2.644	7.545	1	0.006
Age[3]	-0.383	0.273	-0.946	0.138	2.054	1	0.152
Sex[4]	-0.268	0.518	-1.305	0.745	0.270	1	0.604

Notes: reference categories: [1]caregiver; [2]vet check; [4]female; [3]centered and standardized to a mean of zero and a standard deviation of 1.

Table S5. Results of binomial GLM of dogs' blocking response in the Reaction phase.

	Estimate	SE	95% CI		χ^2	df	P
(Intercept)	-0.305	0.414	-1.132	0.503			
Human[1]	-1.043	0.431	-1.910	-0.214	6.111	1	0.013
Treatment[2]	0.325	0.434	-0.519	1.189	0.567	1	0.451
Age[3]	-0.407	0.225	-0.865	0.022	3.456	1	0.063
Sex[4]	0.525	0.429	-0.311	1.378	1.514	1	0.219

Notes: reference categories: [1]caregiver; [2]vet check; [4]female; [3]centered and standardized to a mean of zero and a standard deviation of 1.

Table S6. Results of negative-binomial GLM of dogs' sniffing of fake dog's anal region response in the Reaction phase.

	Estimate	SE	95% CI		χ^2	df	P
(Intercept)	0.783	0.170	0.449	1.112			
Human[1]	-0.418	0.173	-0.759	-0.079	5.841	1	0.016
Treatment[2]	0.203	0.174	-0.138	0.545	1.357	1	0.244

	Estimate	SE	95% CI		χ^2	df	P
Age[3]	-0.162	0.089	-0.342	0.016	3.173	1	0.075
Sex[4]	0.181	0.172	-0.157	0.520	1.101	1	0.294

Notes: reference categories: [1]caregiver; [2]vet check; [4]female; [3]centered and standardized to a mean of zero and a standard deviation of 1.

Table S7. Results of binomial GLM of dogs' friendly interaction with human response in the Reaction phase.

	Estimate	SE	95% CI		χ^2	df	P
(Intercept)	-0.609	0.417	-1.454	0.196			
Human[1]	-0.809	0.430	-1.670	0.022	3.640	1	0.056
Treatment[2]	0.698	0.435	-0.144	1.571	2.634	1	0.105
Age[3]	-0.183	0.220	-0.626	0.244	0.698	1	0.403
Sex[4]	0.121	0.428	-0.720	0.965	0.080	1	0.777

Notes: reference categories: [1]caregiver; [2]vet check; [4]female; [3]centered and standardized to a mean of zero and a standard deviation of 1.

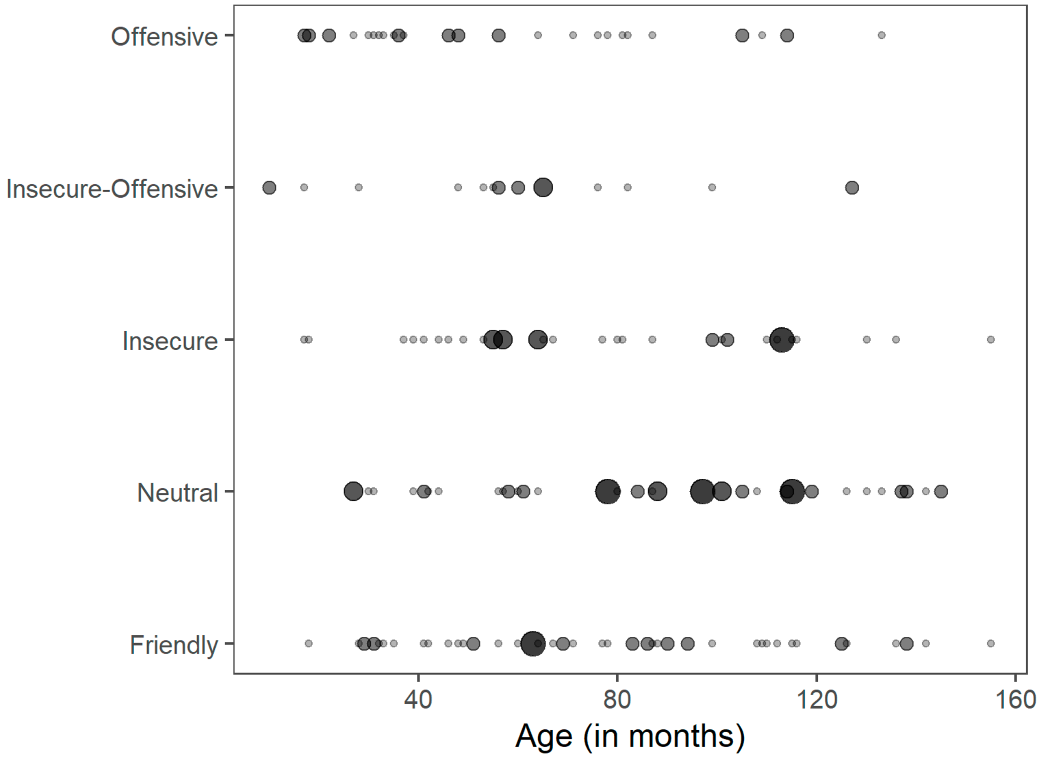

Figure S1. Behavioural ratings in the Introduction and Interaction phases as a function of dogs' age (in months). The size of the dots is proportional to the number of represented data points.
